# Analysis of COVID-19 vaccine non-intent by essential vs non-essential worker, demographic, and socioeconomic status among 101,048 US adults

**DOI:** 10.1371/journal.pone.0258540

**Published:** 2021-10-28

**Authors:** Tania Elliott, Baligh R. Yehia, Angela L. Winegar, Jyothi Karthik Raja, Ashlin Jones, Erin Shockley, Joseph Cacchione

**Affiliations:** 1 Clinical and Network Services, Ascension Health, Saint Louis, Missouri, United States of America; 2 Ascension Data Science Institute, Saint Louis, Missouri, United States of America; University of Arkansas for Medical Sciences, UNITED STATES

## Abstract

As of May 2021, over 286 million coronavirus 2019 (COVID-19) vaccine doses have been administered across the country. This data is promising, however there are still populations that, despite availability, are declining vaccination. We reviewed vaccine likelihood and receptiveness to recommendation from a doctor or nurse survey responses from 101,048 adults (≥18 years old) presenting to 442 primary care clinics in 8 states and the District of Columbia. Occupation was self-reported and demographic information extracted from the medical record, with 58.3% (n = 58,873) responding they were likely to receive the vaccine, 23.6% (n = 23,845) unlikely, and 18.1% (n = 18,330) uncertain. We found that essential workers were 18% less likely to receive the COVID-19 vaccination. Of those who indicated they were not already “very likely” to receive the vaccine, a recommendation from a nurse or doctor resulted in 16% of respondents becoming more likely to receive the vaccine, although certain occupations were less likely than others to be receptive to recommendations. To our knowledge, this is the first study to look at vaccine intent and receptiveness to recommendations from a doctor or nurse across specific essential worker occupations, and may help inform future early phase, vaccine rollouts and public health measure implementations.

## Introduction

Since December 2020, the United States (U.S.) Food and Drug Administration has issued three emergency use authorizations for coronavirus 2019 (COVID-19) vaccines. As of May 2021, over 286 million doses have been administered across the country, with over 39 percent of the US population fully vaccinated [1]. This data is promising, however there are still populations that, despite availability, do not intend to be vaccinated.

Earlier studies indicated that perceptions toward accepting COVID-19 vaccination appear to differ based on age, sex, race/ethnicity, socioeconomic status, and political affiliation [1–4]. Additionally, a recent Kaiser Family Foundation survey found slower vaccine uptake among essential workers. As of mid-March, roughly half (48%) of essential workers reported they had already received at least one dose of the COVID-19 vaccine or would get a vaccine as soon as they could. This statistic, however, is more than 20 percent lower than workers employed in other professions or those without jobs (67 and 69 percent, respectively), despite the fact that many states prioritized availability of vaccines to essential workers early on. Additionally, 1 in 5 frontline workers surveyed said they will “definitely not” get vaccinated [5].

Essential workers are a critically important population to vaccinate. Selden et al recently estimated that 123 million adults meet the main CDC increased risk guidelines for severe COVID-19 infection and that up to 74 million increased-risk US adults either live with or are themselves essential workers, which the authors report is likely an underestimate [6]. After adjusting for baseline demographic, socioeconomic, health, and lifestyle-related risk factors, Mutambudzi et al noted that essential workers have a higher risk for severe COVID-19, and that this risk is higher in non-white essential workers [7]. It is becoming increasingly more apparent that additional data is needed to understand what factors are contributing to vaccine non-intent, particularly in populations at highest risk of infection and transmission by nature of their occupation and inability to work from home. An understanding of vaccine intent during the early phases of rollout may inform future public health communication strategies.

## Methods

We recruited a convenience sample of adults (≥18 years old) presenting to 442 primary care clinics in 8 states and the District of Columbia between January 11, 2021 and February 22, 2021 to participate in a survey of vaccine intent and hesitancy developed by Phreesia and the CONVINCE USA initiative at the CUNY Graduate School of Public Health & Health Policy. Clinics were located in urban, suburban, and rural areas within Alabama (58), Florida (29), Indiana (142), Maryland (6), Michigan (117), New York (10), Tennessee (29), Texas (46), and the District of Columbia (5) which serve a population of essential and non-essential workforce comparable to the national distribution [8]. While digitally checking in for their visit, participants were invited to complete a 10-question survey in English or Spanish using their personal device or a tablet available from the physician’s office. Patients provided their current COVID-19 vaccination status, likelihood to receive a COVID-19 vaccine, and self-reported clinical, occupational, and social risk factors for severe COVID-19 illness. Respondents rated their likelihood to accept vaccination using a five-point Likert scale (very unlikely, somewhat unlikely, uncertain, somewhat likely, and very likely). After rating their likelihood to vaccinate, respondents were asked to rate their likelihood to accept vaccination if it were recommended by a healthcare professional using the same five-point Likert scale. Each patient was surveyed only once during the timeframe studied. This study was approved with a waiver of informed consent by the Ascension St. Vincent’s institutional review board.

Analysis was conducted with the primary outcome of intent to receive the COVID-19 vaccine and the secondary outcome of the influence of nurses and doctors on intent to vaccinate. Incorporating factors shown to be important in vaccine willingness, patient-level demographic, social, and clinical characteristics, as well as essential-worker status were included as covariates in the analysis [1, 3, 5]. Demographic characteristics of age, gender, race, and health insurance status were abstracted from the medical record. Documentation of ethnicity in the medical record was incomplete and therefore not included and small sample sizes for races other than White and Black precluded their inclusion. Using each patient’s home address and census tract, we assigned the CDC Social Vulnerability Index (SVI), a measure of social vulnerability based on fifteen social factors. As part of the survey, individuals self-reported COVID-19 risk factors, including medical conditions placing them at increased risk of severe COVID-19 illness, residence in a nursing home or assisted living facility, and employment as an essential worker (following the CDC’s criteria for essential worker status) [9]. Because some survey respondent groups were relatively small (e.g., nursing home residents), a Bayesian logistic regression was chosen to provide intuitive characterizations of uncertainty in effect estimates while evaluating associations between patient characteristics and likelihood to be vaccinated by calculating adjusted odds ratios with 95% credible intervals (CIs). The same approach was applied to assess the association between patient characteristics and the degree to which doctors and nurses are able to influence intent to receive a COVID-19 vaccination, specifically among patients who had indicated they were not already “very likely” to accept vaccination. To descriptively characterize the spectrum of intention of essential worker responses to receive the COVID-19 vaccine, vaccine non-intent and nurse/doctor vaccine recommendation response were plotted for each essential worker category, with results presented as z-score normalized values. Statistical analyses were performed using Python version 3.9.2 and the Python package PyMC3 version 3.11.1.

## Results

**Fig 1:**
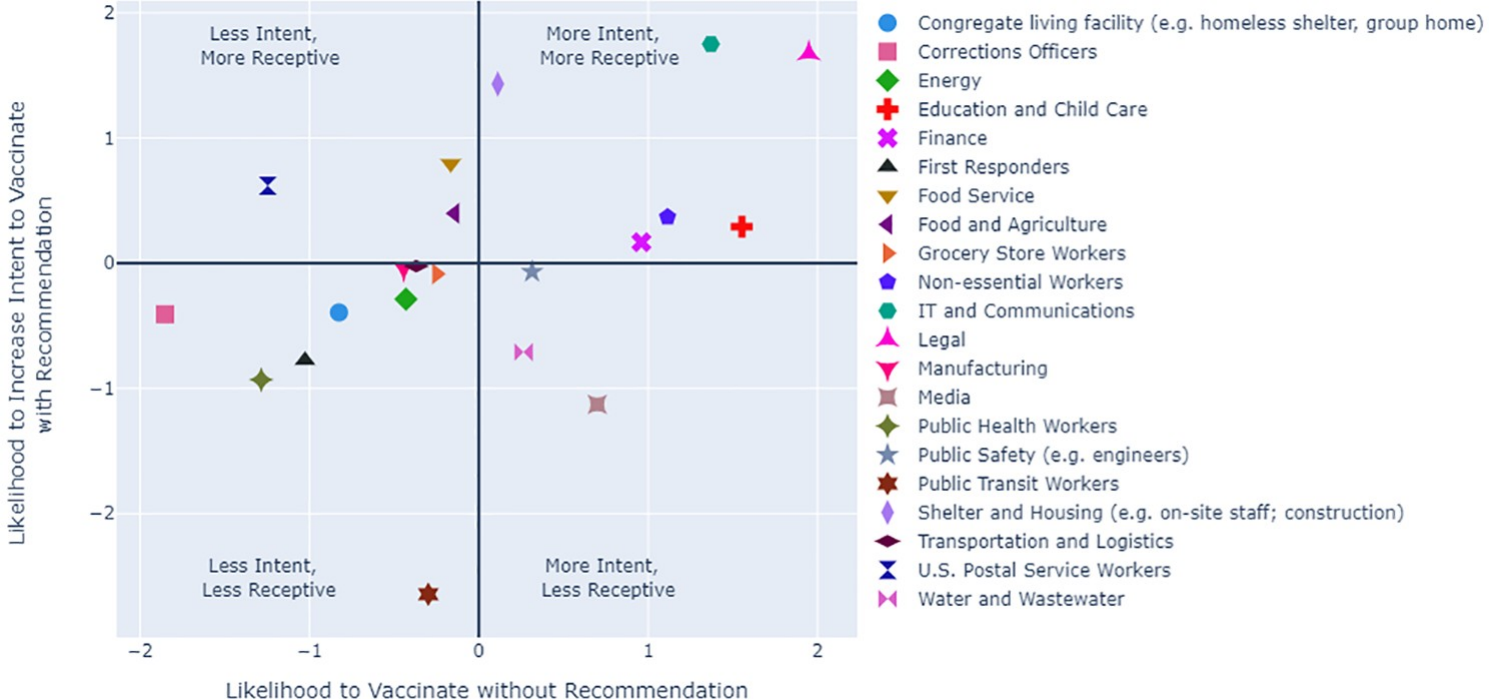
Likelihood to vaccinate vs change in vaccine intent with healthcare professional vaccine recommendation for essential worker classes.

It is estimated that 20–25% of patients seen in our primary care settings during the period of January 11 to February 22 completed the survey evaluating their intent to receive the COVID-19 vaccination, however response rate in the individual clinics ranged from 1% to 98%. Of the 118,873 surveys completed, 17,825 (15.0%) indicated that they had already received at least one vaccine dose and were excluded. As shown in Table 1, of the remaining 101,048 individuals, 77.4% (78,163) were white and 64.5% (65,207) female. The median age was 50.9 years. 58.3% (n = 58,873) responded they were likely to receive the vaccine, 23.6% (n = 23,845) unlikely, and 18.1% (n = 18,330) uncertain. Essential workers were less likely to respond “very/somewhat likely” (52.3%; n = 19,449) to receive the vaccine than non-essential workers (61.8%; n = 39,424). Among essential workers, corrections officers had the highest percentage of “very/somewhat unlikely” (41.3%), followed by public health workers (37.5%), and US postal service workers (35.9%), representing 0.3%, 5%, and 0.5% of the population; respectively. Of all respondents who were not already very likely to vaccinate, 16% stated they would be more likely to vaccinate if a doctor or nurse recommended the vaccine. The degree to which doctors and nurses influenced different essential worker categories ranged from 6.8% to 20.9% (Table 1). Along with US postal service workers, food and agriculture workers and food service workers were also among the most hesitant categories of essential workers who indicated they would increase their receptiveness to a COVID-19 vaccination following a recommendation from the medical community (Fig 1).

**Table 1:** Demographic representation of survey respondents who indicated they had not yet received a COVID-19 vaccine.

			Likelihood to Vaccinate			Influence of Doctor/Nurse Recommendation on Likelihood to Vaccinate
Characteristic		% (n)	% Very/ Somewhat Likely	% Uncertain	% Very/ Somewhat unlikely	Of those not already "Very Likely"—% who increased at least one box
**Total**		100% (101,048)	58.3% (58,873)	18.1% (18,330)	23.6% (23,845)	15.8% (8,734)
**Gender**						
	Female	64.5% (65,207)	55.0% (35,855)	19.4% (12,661)	25.6% (16,691)	15.4% (5,830)
	Male	35.5% (35,841)	64.2% (23,018)	15.8% (5,669)	20.0% (7,154)	16.7% (2,904)
**Race**						
	White	77.4% (78,163)	60.7% (47,474)	16.2% (12,642)	23.1% (18,047)	16.7% (6,753)
	Black	13.9% (14,031)	44.8% (6,289)	25.7% (3,612)	29.4% (4,130)	12.3% (1,203)
	Other Race	3.0% (3,010)	50.1% (1,509)	27.6% (831)	22.3% (670)	14.4% (272)
	Patient Declined	3.6% (3,669)	53.4% (1,958)	24.1% (886)	22.5% (825)	14.7% (322)
	Asian	2.2% (2,175)	75.5% (1,643)	16.5% (359)	8.0% (173)	20.7% (184)
**Age**						
	18–49	48.0% (48,483)	46.8% (22,687)	21.9% (10,601)	31.3% (15,195)	13.9% (4,557)
	50–64	31.6% (31,882)	64.1% (20,433)	16.7% (5,328)	19.2% (6,121)	17.7% (2,750)
	65–74	15.0% (15,196)	75.8% (11,522)	11.5% (1,748)	12.7% (1,926)	20.5% (1,054)
	75+	5.4% (5,487)	77.1% (4,231)	11.9% (653)	11.0% (603)	21.9% (373)
**In a Nursing Home or Assisted Living Facility**						
	No	99.5% (62,550)	59.4% (37,155)	18.1% (11,294)	22.5% (14,101)	15.8% (8,693)
	Yes	.47% (294)	60.3% (259)	18.6% (80)	21.0% (90)	18.7% (41)
**Has 1+ medical COVID risk factor** *						
	No	53.5% (54,026)	56.6% (30,573)	19.1% (10,303)	24.3% (13,150)	14.6% (4,477)
	Yes	46.5% (47,022)	60.2% (28,300)	17.1% (8,027)	22.7% (10,695)	17.4% (4,257)
**Is an essential worker**						
	No	63.2% (63,828)	61.8% (39,424)	17.3% (11,036)	20.9% (13,368)	16.6% (5,330)
	Yes	36.8% (37,220)	52.3% (19,449)	19.6% (7,294)	28.1% (10,477)	14.8% (3,404)
**Essential worker job category**						
	Congregate living facility	0.4% (372)	44.6% (166)	22.8% (85)	32.5% (121)	13.8% (34)
	Corrections officers	0.3% (298)	35.6% (106)	23.2% (69)	41.3% (123)	12.2% (28)
	Education or child care	5.5% (5,553)	65.6% (3,642)	14.6% (810)	19.8% (1,101)	17.8% (481)
	Energy	0.6% (609)	48.1% (293)	18.2% (111)	33.7% (205)	13.6% (55)
	Finance	2.4% (2,419)	60.4% (1,460)	16.3% (395)	23.3% (564)	16.2% (218)
	First responders	1.6% (1,652)	42.9% (708)	21.5% (355)	35.7% (589)	13.0% (153)
	Food and agriculture	1.9% (1,937)	50.6% (981)	21.6% (418)	27.8% (538)	15.1% (183)
	Food service	2.3% (2,278)	50.4% (1,149)	23.0% (525)	26.5% (604)	16.7% (248)
	Grocery store workers	2.3% (2,285)	49.7% (1,136)	22.8% (520)	27.5% (629)	14.3% (211)
	IT and Communications	1.5% (1,549)	64.0% (991)	15.6% (241)	20.5% (317)	18.0% (141)
	Legal	.7% (734)	69.1% (507)	12.9% (95)	18.0% (132)	20.9% (66)
	Manufacturing	4.9% (4,992)	48.0% (2,396)	21.3% (1,063)	30.7% (1,533)	13.5% (439)
	Media	.2% (248)	58.1% (144)	21.8% (54)	20.2% (50)	13.1% (16)
	Public safety (e.g. engineers)	.7% (664)	54.7% (363)	17.9% (119)	27.4% (182)	15.8% (63)
	Public health workers	5.0% (5,086)	40.6% (2,064)	22.0% (1,119)	37.4% (1,903)	12.4% (465)
	Public transit workers	.3% (276)	49.3% (136)	23.9% (66)	26.8% (74)	6.8% (12)
	Shelter and Housing	1.5% (1,505)	52.9% (796)	18.9% (285)	28.2% (424)	16.9% (160)
	Transportation and Logistics	2.8% (2,806)	48.6% (1,365)	20.9% (586)	30.5% (855)	14.5% (262)
	U.S. Postal service workers	.5% (496)	40.9% (203)	23.2% (115)	35.9% (178)	14.0% (51)
	Water and Wastewater	.4% (402)	54.2% (218)	19.2% (77)	26.6% (107)	14.7% (34)
**Payer**						
	Commercial	58.2% (58,797)	60.7% (35,715)	16.8% (9,869)	22.5% (13,213)	16.2% (5,084)
	Medicare	16.3% (16,482)	71.3% (11,755)	13.5% (2,225)	15.2% (2,502)	19.7% (1,256)
	Medicaid	13.7% (13,815)	37.7% (5,205)	27.1% (3,748)	35.2% (4,862)	12.7% (1,295)
	Other	11.8% (11,954)	51.8% (6,198)	20.8% (2,488)	27.3% (3,268)	14.9% (1,099)
**Overall SVI** **						
	0–0.25 Lowest Vulnerability	30.8% (31,100)	68.9% (21,433)	13.8% (4,281)	17.3% (5,386)	16.9% (2,316)
	0.26–0.50 Low-to-Mid Vulnerability	30.2% (30,533)	57.9% (17,679)	17.8% (5,441)	24.3% (7,413)	16.0% (2,688)
	0.51–0.75 Mid-to-High Vulnerability	23.3% (23,516)	52.4% (12,328)	20.4% (4,803)	27.2% (6,385)	15.7% (2,225)
	0.76–1.00 Highest Vulnerability	15.7% (15,899)	46.8% (7,433)	23.9% (3,805)	29.3% (4,661)	14.4% (1,505)
**Vaccine Rollout Phase** ***						
	1a	6.7% (6,753)	46.3% (3,124)	20.3% (1,372)	33.4% (2,257)	13.3% (613)
	1b	21.4% (21,575)	59.4% (12,822)	17.7% (3,812)	22.9% (4,941)	15.9% (1,814)
	1c	46.9% (47,433)	61.0% (28,917)	16.9% (8,015)	22.1% (10,501)	17.1% (4,159)
	Later phase	25.0% (25,287)	55.4% (14,010)	20.3% (5,131)	24.3% (6,146)	14.5% (2,148)

* medical risk factors include: ’Cancer’, ’Chronic Kidney Disease’, ’COPD’, ’Down Syndrome’, ’Heart Conditions’, ’Immunocompromised State’, ’Obesity’, ’Pregnancy’, ’Sickle Cell Disease’, ’Smoking’, ’Diabetes’

** patient address mapped to CDC 2018 Social Vulnerability Index (SVI)

*** Calculated from CDC guidelines, age, essential worker type, and medical risk factors

Adjusting for social, demographic, and medical characteristics, factors associated with increased likelihood to vaccinate include age and the presence of a medical risk factor for severe COVID-19. Respondents 75 years and older were 3.4 times as likely to vaccinate as respondents aged 18–49. Individuals with COVID-19 risk factors were 10% more likely to vaccinate. Results differed by race; compared to Whites, Black/African Americans were 30% less likely to vaccinate while Asians were twice as likely. Both Medicaid and the most socially vulnerable patients were half as likely to vaccinate compared to commercially insured and least vulnerable patients, respectively. Respondents who identified as essential workers were 18% less likely than non-essential workers (Table 2).

**Table 2:** Results of a Bayesian regression analysis, adjusted for demographic/clinical, occupational, and social risk factors, on response of "very/somewhat likely to vaccinate".

Factor		Adjusted OR (95% CI)	Probability of OR excluding 1*
**Demographic and clinical factors**			
***Sex***			
	Female	1 [Reference]	
	Male	1.306 (1.271–1.343)	> = 0.95
***Age Group***			
	18–49	1 [Reference]	
	50–64	1.844 (1.789–1.904)	> = 0.95
	65–74	3.169 (3.001–3.329)	> = 0.95
	75+	3.392 (3.145–3.647)	> = 0.95
***Race***			
	White	1 [Reference]	
	Asian	2.210 (1.987–2.441)	> = 0.95
	Black or African American	0.715 (.686-.744)	> = 0.95
	Other	0.822 (.759-.886)	> = 0.95
	Patient Declined	0.869 (.808-.930)	> = 0.95
***Payer***			
	Commercial payer	1 [Reference]	> = 0.95
	Medicaid	0.480 (.460-.500)	> = 0.95
	Medicare	0.830 (.786-.869)	> = 0.95
	Other	0.737 (.707-.768)	> = 0.95
***Healthcare System Service Area***			
	Indianapolis/Evansville, IN	1 [Reference]	
	Birmingham/Mobile, AL	0.781 (.750-.813)	> = 0.95
	Washington, DC	1.554 (1.34–1.779)	> = 0.95
	Jacksonville/Pensacola, FL	0.811 (.772-.852)	> = 0.95
	Baltimore, MD	2.073 (1.842–2.312)	> = 0.95
	Detroit/Kalamazoo, MI	1.063 (1.024–1.104)	> = 0.95
	Binghamton, NY	1.148 (1.070–1.226)	> = 0.95
	Nashville, TN	0.968 (.906–1.031)	0.842
	Austin/Waco, TX	1.158 (1.102–1.215)	> = 0.95
***COVID-19 Medical Risk Factors***			
	None	1 [Reference]	
	One or more	1.102 (1.073–1.132)	> = 0.95
**Occupational factor**			
	Not an essential worker	1 [Reference]	
	Essential worker	0.826 (.801-.849)	> = 0.95
**Social factors**			
***Nursing home/assisted living status***			
	Not a nursing home/assisted living resident	1 [Reference]	
	Resides in nursing home/assisted living	1.166 (.935–1.416)	0.922
***CDC 2018 Social Vulnerability Quartile***			
	Lowest vulnerability	1 [Reference]	
	Low-Mid vulnerability	0.655 (.632-.678)	> = 0.95
	Mid-High vulnerability	0.564 (.543-.585)	> = 0.95
	Highest vulnerability	0.516 (.493-.539)	> = 0.95

* A > = 0.95 probability of the OR excluding 1 indicates reasonable confidence of the presence of an effect given the data available and is provided for comparison to frequentist null hypothesis significance testing.

Statistical analyses were performed using Python version 3.9.2 and the Python package PyMC3 version 3.11.1. The intercept was given a flat prior distribution. All regression coefficients were given a weakly informative Cauchy prior with α = 0 and β = 2.5, as suggested in Gelman 2008. MCMC chain convergence was assessed using the Gelman-Rubin convergence criterion; all were less than 1.05.

Among the subset of respondents who indicated they were not already “very likely” to receive a COVID-19 vaccine and adjusting for social, demographic, and medical characteristics, we identified populations most and least influenced by nurse or doctor recommendation. Respondents most influenced were those over age 75 years (1.5 times more likely to become more accepting of a COVID-19 vaccine compared with respondents aged 18–49), Asians (40% more likely than Whites), and individuals with one or more COVID-19 risk factors (20% more likely than those without risk factors). Several other populations responded that a recommendation from a nurse or a doctor was unlikely to influence their intent to vaccinate, compared to the reference group. Essential workers (6% less likely), Black/African Americans (25% less likely), Medicaid recipients (20% less likely), and those classified by the CDC as socially vulnerable (8% less likely) were among the populations least likely to increase their vaccine intent following a medical recommendation, compared to non-essential workers, Whites, Commercial insurance beneficiaries and those classified by the CDC as least socially vulnerable, respectively (Table 3).

**Table 3:** Results of a Bayesian regression analysis, adjusted for demographic/clinical, occupational, and social risk factors, on increased likelihood to vaccinate after doctor/nurse recommendation.

Factor		Adjusted OR (95% CI)	Probability of OR excluding 1*
**Demographic and clinical factors**			
***Sex***			
	Female	1 [Reference]	
	Male	1.062 (1.008–1.114)	> = 0.95
***Age Group***			
	18–49	1 [Reference]	
	50–64	1.237 (1.170–1.301)	> = 0.95
	65–74	1.393 (1.267–1.520)	> = 0.95
	75+	1.497 (1.294–1.698)	> = 0.95
***Race***			
	White	1 [Reference]	
	Asian	1.391 (1.161–1.624)	> = 0.95
	Black or African American	0.748 (.696-.802)	> = 0.95
	Other	0.854 (.745-.972)	> = 0.95
	Patient Declined	0.916 (.808–1.032)	0.924
***Payer***			
	Commercial payer	1 [Reference]	
	Medicaid	0.801 (.745-.858)	> = 0.95
	Medicare	0.988 (.905–1.075)	> = 0.95
	Other	0.909 (.845-.974)	> = 0.95
***Healthcare System Service Area***			
	Indianapolis/Evansville, IN	1 [Reference]	
	Birmingham/Mobile, AL	1.044 (.976–1.117)	0.893
	Washington, DC	1.350 (1.043–1.699)	> = 0.95
	Jacksonville/Pensacola, FL	0.980 (.894-.1.059)	0.688
	Baltimore, MD	1.127 (.906–1.353)	>0.863
	Detroit/Kalamazoo, MI	0.905 (.842-.964)	> = 0.95
	Binghamton, NY	0.947 (.831–1.068)	0.812
	Nashville, TN	1.264 (1.135–1.406)	> = 0.95
	Austin/Waco, TX	1.203 (1.099–1.303)	> = 0.95
***COVID-19 Medical Risk Factors***			
	None	1 [Reference]	
	One or more	1.208 (1.150–1.263)	> = 0.95
**Occupational factor**			
	Not an essential worker	1 [Reference]	
	Essential worker	.934 (.888-.979)	> = 0.95
**Social factors**			
***Nursing home/assisted living status***			
	Not a nursing home/assisted living resident	1 [Reference]	
	Resides in nursing home/assisted living	1.268 (.859–1.726)	0.890
***CDC 2018 Social Vulnerability Quartile***			
	Lowest vulnerability	1 [Reference]	
	Low-Mid vulnerability	0.943 (0.886–1.004)	> = 0.95
	Mid-High vulnerability	0.942 (0.882–1.006)	> = 0.95
	Highest vulnerability	0.920 (0.852–0.991)	> = 0.95

* A > = 0.95 probability of the OR excluding 1 indicates reasonable confidence of the presence of an effect given the data available and is provided for comparison to frequentist null hypothesis significance testing.

Statistical analyses were performed using Python version 3.9.2 and the Python package PyMC3 version 3.11.1. The intercept was given a flat prior distribution. All regression coefficients were given a weakly informative Cauchy prior with α = 0 and β = 2.5, as suggested in Gelman 2008. MCMC chain convergence was assessed using the Gelman-Rubin convergence criterion; all were less than 1.05.

## Discussion

To our knowledge, this is the first study to look at vaccine intent and receptiveness to recommendations from a doctor or nurse across specific essential worker occupations. We found that certain front-line workers who were already vaccine hesitant were less receptive to vaccination than other occupations after a recommendation from a doctor or nurse. This included both public health workers and first responders. Further study is required to understand why individuals, who presumably have seen the impacts of COVID-19 firsthand, would be less likely to vaccinate. Perhaps there is an opportunity to explore whether likelihood to vaccinate would change if the recommendation came from their trusted healthcare professional, such as another colleague or a patient’s own physician, or, if it is more effective to receive messaging from a thought leader outside of healthcare. It is also important to recognize that there may be an inherent distrust of the healthcare system for certain populations, and that reasons for vaccine non-intent may be multifactorial and driven by other factors aside from occupation, including political partisanship, cultural beliefs and norms, and general healthcare attitudes and practices [3]. We need to be cautious about overgeneralization of what type of messaging is most effective.

Other findings were generally consistent with prior research [1, 7]. As has been previously reported, we found that non-intent is still high in African Americans and socially vulnerable populations, suggesting that vaccine engagement approaches in these populations should be revisited. Prior studies also suggest that vaccine likelihood among healthcare workers is increasing, ranging from only 30 percent in the early stages of rollout, to over 70 percent in recent weeks [10]. Still, worldwide, vaccine skepticism among healthcare workers remains, with individuals citing concerns around safety, efficacy, and side effects [11, 12]. Prior research also suggests that overall, patients’ likelihood to vaccinate increases with a recommendation from a healthcare professional [13], which is also consistent with our overall findings, however, our analysis brings new insight in that this was not applicable essential workers. This finding may inform future public health measure or vaccine rollout strategies.

This study is limited by use of an English and Spanish convenience sample with a response rate of 20–25% and that our analysis excluded those already vaccinated. With a convenience sample, it can be difficult to generalize the findings; however, the primary care clinic setting is the ideal location to assess the impact of nurse and doctor recommendations on vaccine intent, since that is where many vaccines have historically been administered. Additionally, our sample size is large, varied, and representative of our overall patient population in the previous year, and includes a broad geographic distribution of primary care clinics in rural, suburban, and urban areas. Additionally, and important to our findings, the sample consists of a proportional distribution of essential and non-essential workers, as is found in the national adult population [8]. Our study is also limited in that it does not reflect possible changes in attitudes over time, and specifically addresses intent to vaccinate, which may be different from actual behavior. The survey was conducted in the earlier phases of vaccine rollout, so may not be reflective of patients who could have been impacted by campaigns addressing vaccine effectiveness or lack of information or scientific consensus. Additionally, we used the CDC’s definition of essential worker [4]. While this list is generally accepted for purposes of vaccine prioritization, the definitions have not been adopted by all 50 states.

Our findings highlight that essential workers are a heterogeneous group regarding vaccine intent. Of the essential workers who were not already very likely to vaccinate, 16 percent were influenced by a doctor or nurse recommendation. One approach to addressing the remaining population is to identify thought leaders within occupations, such as union leaders, to serve as advocates for vaccination. Another is to consider a test and learn approach to different kinds of messaging across multiple populations, similar to what is done in consumer focused industries. Ultimately, further research is needed to identify other factors associated with non-intent to better segment our populations, identify and address the root cause of concern(s), and identify champions outside of healthcare who can create messaging that will resonate with their audience.
